# To What Extent Are Consumers’ Perception and Acceptance of Alternative Meat Production Systems Affected by Information? The Case of Cultured Meat

**DOI:** 10.3390/ani10040656

**Published:** 2020-04-10

**Authors:** Maria Cecilia Mancini, Federico Antonioli

**Affiliations:** Dipartimento di Scienze Economiche e Aziendali, Università degli Studi di Parma, 43125 Parma, Italy; federico.antonioli@unipr.it

**Keywords:** cultured meat, willingness to pay, information, consumer perception, animal welfare, novel food

## Abstract

**Simple Summary:**

Meat grown in labs, also known as cultured meat, is currently under development and will likely soon be available on supermarket shelves. Such new meat-based products may tackle some of the most controversial societal concerns related to the industry, in particular animal wellbeing and environmental impacts, with further potential improvements concerning food security. However, due to its high degree of novelty, it remains unclear how consumers view this type of food product, particularly in terms of beliefs regarding its intrinsic attributes such as safety, nutrients and flavor characterization, and its positive externalities concerning the environment, animal welfare, and food security. The present study aims at unveiling the perception, acceptance, and willingness to try, buy, and pay a premium price for cultured meat in the Italian context, deconvoluting the effect of providing positive information to consumers. Such investigation offers new insights for the development of targeted marketing strategies by deepening the understanding of consumers’ perception of this lab-grown food product. Indeed, the study reveals that positive information affects the consumers’ perception towards safety and nutritional characteristics of cultured meat and the willingness to pay a premium price for this new food product accordingly.

**Abstract:**

The global meat production system is currently under pressure, particularly for its environmental and animal wellbeing impacts, as well as for the increasing protein demand worldwide. In this regard, cultured meat is currently a hot topic in the industrial, political, and societal arenas, revealing itself as the potential relief for the issues above. However, its high degree of novelty may hamper the extent of consumers’ acceptance. This research assesses for which beliefs concerning intrinsic attributes and positive externalities, the provision of information is a sufficient tool for affecting the perception and acceptance of cultured meat on a panel of Italian consumers. Changes in perception and willingness to try, buy, and pay are assessed by measuring the variation before and after the provision of positive information related to the product. The results show that perception is affected by positive information concerning safety and nutritional characteristics, whereas the opposite occurs regarding the product flavor. Furthermore, findings reveal that, while the willingness to buy increases after providing positive information, the willingness to try does not. Finally, information on intrinsic attributes and positive externalities of the cultured meat would have to be combined with different approaches for further enhancement of consumers’ perception and acceptance.

## 1. Introduction

At present, the meat production system is under pressure because of several concurrent factors. According to [1], 815 million hungry people require nourishment worldwide and an additional 2 billion people are expected to be added to this total by 2050. In addition, urbanization, population growth, and rising incomes in developing countries will increase the global demand for animal products by more than two-thirds by 2050 [1]. At the same time, Western consumers appear to be unwilling to substantially reduce meat consumption [2], suggesting one should expect a significant rise in livestock production [3], resulting in a significant negative impact on natural resources. Indeed, animal production generates approximately 14.5% of the total anthropogenic greenhouse-gas emissions [4], and, despite an increased efficiency regarding production processes, the expected growth in demand will probably enlarge this share in the coming decades. Thus, viable actions are needed for enabling the food system [5] to mitigate the increasing demand for protein sources and both social and environmental priorities, such as animal welfare [6,7,8,9,10,11], particularly in animal transportation [12], providing a potential solution to the so-called ‘meat paradox’ (this term relates to the current phenomenon where “one believes in the value of animal wellbeing and nonetheless consumes products which have caused animal suffering” [13]). One potential food scenario is represented by cultured meat, which is produced by using animal cells taken from a living animal and then grown in a laboratory environment, exercised, and nourished with a nutrient-rich serum [14,15,16].

Cultured meat, also known as in vitro, synthetic meat, clean meat and by a number of more or less widespread terms [17], is currently a hot topic in both the policy and industrial agendas: the European Union legislator is working to provide a legislative framework to protect consumers (e.g., the Reg. (EU) 2015/2283 on novel foods) [18], whereas food industry players and investors are financing scientific research to accelerate the availability of the product to the consumer market.

The recent literature focuses on the positive environmental effects of cultured meat production compared to conventional meat production. In an early publication, cultured meat was indicated to reduce energy consumption and land usage by 99%, water usage by 90%, and energy consumption by 40% [19]; however, a higher energy input and water footprint was reported in later research [20]. More recently, some authors have reported scaled-down performances of cultured meat, resulting from having a lower land footprint than beef and lower greenhouse gas emissions than poultry, pork, and beef, but a higher energy consumption than pork and poultry and comparable to beef [21]. The literature has identified other benefits related to cultured meat: it would largely circumvent the need for animals in the meat production system, thus alleviating ethical concerns of meat-eaters associated with industrial livestock operations [22,23] and address global hunger issues, avoiding meat becoming a luxury food for the majority of the consumers [15]. The controlled environment of the production process would also allow for a reduction in human–animal interactions, thereby reducing the risks of zoonosis and other diseases [15,24]. Nevertheless, some authors warn against both the cell culture, which is never entirely controlled, and some unexpected biological mechanisms that could occur [25,26].

The commercial success of cultured meat, however, heavily depends on consumer perception. Bryant and Barnett [27] offer a thorough review article on consumers’ perception towards cultured meat, together with some other very recent studies on this topic [28,29,30,31]. Common objections to cultured meat relate to either personal or societal concerns. The former relates to safety issues [26,32,33,34,35], the nutritional value [32,33,36,37,38], and the taste/texture/appearance of cultured meat [32,33,37,38,39,40,41,42], and some others call for more in-depth investigations to gain a better understanding of consumer profiles [43]. The latter mainly pertains to the end of traditional animal agriculture, distrust in the companies producing cultured meat, and the energy required for production [32,33,37,38,40]. A positive consumers’ perception of cultured meat is mainly related to animal welfare and environmental sustainability [32,33,44,45]—less frequently to food security and safety [33,37,38,39,44].

An essential element related to consumer acceptance is information provisioning, as shown by some empirical analyses [33,38,40,46] that prove that the higher the consumers’ familiarity with cultured meat, the higher their acceptance rate. Conversely, the higher the general aversion to a new taste and food experience, the lesser the willingness of the consumers’ to eat cultured meat, resulting in a lesser acknowledgement of its benefits [30]. Information, particularly regarding the environmental benefits, results in being an essential medium to address Belgians’ positive perception of cultured meat [37]. Not only did the themes and sources of information prove to be critical, but also the wording and metaphors represent a useful tool that influenced consumer opinion [47]. In this regard, Bryant and Barnett [27] identify a gap in the literature regarding the persuasiveness of emphasizing the different benefits of cultured meat. Nevertheless, Siegrist et al. [35] and Bryant and Dillard [48] reported a significantly higher rate of acceptance when participants are given a non-technical description of cultured meat compared to a technical description due to a difference in perceived naturalness. Information can change the explicit attitude, i.e., the evaluation constructed through the cognitive elaboration of available information [49] towards the unfamiliar object—cultured meat—in the direction of the valence of the information [38]. Nevertheless, solving the “information deficit” may result insufficient to overcome the aversion to novel technologies applied to food production [28,30]. Indeed, the current available literature fails to provide a comprehensive analysis about consumers’ perception towards intrinsic attributes and positive externalities the cultured meat embeds. In particular, as suggested by [28,30], further research is needed to understand whether the consumers’ perception and beliefs are effectively influenced by information related to both intrinsic attributes and positive externalities. This would unveil the attributes and externalities for which approaches other than positive information provision are needed to face psychological barriers, such as distrust and fear.

To the best of authors’ knowledge, the literature lacks studies concerning the role and the limits of positive information on consumers’ perception and acceptance of cultured meat regarding the Mediterranean basin, particularly Italy. 

The present work aims to fill this gap by assessing to what extent Italian consumers’ perception and their willingness to try, buy, and pay a premium price for cultured meat are affected by positive information. Results offer some insights for both the private and public sectors concerning the potential future promotion of its consumption (some preliminary results of this research are available in [50], where a general discussion on consumers’ perception and acceptance towards cultured meat was provided, but the role of information was not analyzed).

## 2. Materials and Methods 

Consumers’ perception is described by the score given to six statements concerning their beliefs related to intrinsic attributes and positive externalities of the production process for cultured meat, while indicators of consumers’ acceptance of cultured meat are assumed to represent their willingness to try, buy, and pay a premium. 

The first research question we aimed to answer is (i) whether consumers’ perception is positively affected by positive information provisioning. Indeed, some studies on novel foods [51,52,53,54,55] found that positive information affects consumers’ responses to novel food positively. Furthermore, the present research aimed at (ii) confirming the role of the positive information provision in increasing acceptance, defined by the three willingness-related items named above, as shown in [37] and other studies focusing on novel and reformulated foods [56,57]. Finally, the results allowed us to draw some qualitative conclusions on (iii) the effect of a change in perception on the level of acceptance towards a novel food such as cultured meat, i.e., whether the change in the former, prompted by information provision, is predictive of a change in the latter. This opens the door to further research on this topic, particularly related to the predictors of acceptance of the product at stake. Indeed, the market failure for many novel foods is still high, mainly due to unfamiliarity with the product and the high risk perceived by consumers [58,59,60,61]. This highlights the importance of understanding how to predict consumer acceptance and behavior towards novel food.

An online survey was conducted, reaching 525 Italian consumers (N = 525) between September 2017 and March 2018. The questionnaire was made available on the website of an Italian consumer association (Confconsumatori), whose main aim is to assist citizens in dealing with public administration issues (in areas such as access to justice) but also with inefficiencies or injustices related to telephone services and tariffs, water, electricity, and gas contracts. 

Thus, as the association is not focused on food issues, the sample is not biased by any specific sensitivity to food-related issues. The members of the association (almost 30,000) were informed about the questionnaire through the Newsletter, which is e-mailed each month, and invited to fill it out.

Respondents that completed the whole questionnaire were rewarded with a free copy of a monthly magazine aimed at informing citizens and consumers on their rights. Once data collection was completed, the e-mails of the respondents were checked to find potential duplicates. Six e-mails were found twice. In such cases, the first questionnaire only was included in the sample. The design of the questionnaire, in line with Verbeke et al. [33], is composed of four sections (Figure S1): the first section collected sociodemographic information, meanwhile, the second section explored meat consumption habits (i.e., whether participants were meat consumers or not, the frequency of meat consumption, intention to reduce meat, and reasons for meat consumption). The third section provided the participants with basic information (Level I) on cultured meat: “Cultured beef is created by harvesting muscle cells from a living cow. Researchers of the Maastricht University (culturedbeef.org) state that cells from a single cow would produce 175 million quarter-pounders. Conventional farming methods would need 440,000 cows. The product should not be confused with meat substitutes like tofu because it is real meat, the same as the one produced according to today’s intensive livestock production system. Cultured meat is not available on the market yet.” A graphic description of the production process was also provided.

The participants were then asked to evaluate six statements on a five-point Likert scale, ranging from “I do not agree at all” (1) to “I completely agree” (5). The first three statements related to beliefs concerning the positive externalities of the cultured meat production process (i.e., the contribution to environmental sustainability, animal welfare, and food security), whereas the remaining three pointed to beliefs concerning intrinsic attributes (i.e., safety, taste, and nutritional content). 

Positive externalities exist if the production of a good or service benefits a third party not directly involved in the market transaction, whereas intrinsic attributes are physical attributes of the product that cannot be modified except by changing the physical characteristics of the product [62,63]. The six statements are phrased in Table 1. Further, three questions related to willingness to try (WTT), buy (WTB), and pay (WTP) for cultured meat, respectively, were then asked. Depending on whether the interviewee expressed a WTT the cultured meat, he/she was asked about his/her WTB and, if positive or uncertain, his/her WTP. Concerning the latter, the reference price for conventional burger meat (€7/kg) was calculated as the mean of burger meat prices surveyed in 24 large retailer outlets scattered throughout Italy. Nine different options were available: whether they were willing to pay a premium price compared to a conventional burger (+10%, +20%, +30%, or an unquantified premium price); if unwilling to pay a premium, the respondent was given the option to pay the same price as a conventional burger or “maybe the same price”; finally, the lower-price options (−10%, −20%, or −30%) were made available for those who expressed either no WTP a premium or the same price. 

In the fourth section, the respondents were provided with more detailed information (Level II) about the positive externalities of cultured meat, namely the environmental, health and food safety benefits: “Two-thirds of agricultural land is used for livestock production, which is responsible for about 18% of the greenhouse gas (GHG) emissions [64,65]. The Food and Agriculture Organization of the United Nations (FAO) estimates that rising pro capita income in developing countries will increase by 70% in 40 years. It was shown [20,66] that cultured meat would lead to a 98.8% reduction of GHG emissions, 99.7% of land use and 94% of water use compared to traditional meat. Cultured meat would also prevent diseases such as mad cow disease and microbiological infections, such as Salmonella, due to less contacts between animals and humans. In addition, the fat composition of the meat can be improved, for example by enriching the meat with omega-3 fatty acids.” (Figure S2) Respondents were then asked again to evaluate the same six statements and their WTT, WTB, and WTP.

### Statistical Methodology

The Statistical Package for Social Science (SPSS) version 25.0 was used to perform the statistical analysis of data (IBM Corp. Released 2017. IBM SPSS Statistics for Windows, Version 25.0, Armonk, NY: IBM Corp). The chi-squared (χ^2^) test was applied for testing independence between gender and meat-consumption habits, finding that non-meat consumers are mainly women (please, see the next section for more details). Both the Shapiro–Wilk and Kolmogorov–Smirnov tests for normality were applied, both rejecting the null of normal distribution of responses for each subgroup in which the sample was segmented. Therefore, due to the non-normal distribution of responses, the non-parametric Wilcoxon signed-rank test was used to obtain an understanding of whether and, if so, how the provision of positive information has an effect on consumer perceptions. Accordingly, the two sets of responses, belonging to the same interviewee, and describing his/her responses before and after the provision of information, were compared.

The sample was segmented according to sociodemographic variables (sex, age, education, place of residence), consumption habits (meat/non-meat consumers, reasons for consuming/non-consuming meat, intention or not to reduce meat consumption, reasons for reducing meat consumption), and according to familiarity or non-familiarity with cultured meat. This latter category was defined according to the interviewee’s answer to the multiple-choice question ‘Have you ever heard about cultured meat before?’. The options were; (1) No (2) Yes, but I do not know what it is about, and (3) Yes, and I know its main characteristics. The respondent was considered familiar with the product when the third option was selected.

Given the wide number of subgroups that were analyzed in the research, multiplicity issues may arise. The latter refers to the inflation of the type I error rate when multiple testing is performed, leading to a higher rate of rejection of the null hypothesis, increasing the risk of false-positive findings [67]. To control for multiplicity issues, we performed the Benjamini–Hochberg correction [68], with a false discovery rate (FDR) of 5%. The multiple comparison correction strongly confirms the previous results. However, given that many of the tested variables are not independent (the Spearman correlation matrix indicates high correlation rates, besides being dependent by construction), we failed to ensure the assumption of independence for correctly and wisely applying the Benjamini–Hochberg correction. Accordingly, a multiple-comparison correction that accounts for test non-independency was applied. Specifically, we followed the correction applied in Cheverud [69], and Nyholt [70], in which the correlation among tested variables is accounted for, and, through eigenvalues’ variance, the number of tests to be used to correct the alpha threshold is given (see Derringer [71] for further details on its implementation in R [72]). Specifically, we determined the precise number of tests (*T*) accounting for k correlated variables as T=1+[(k−1)×(1−var(ϑ)/k)], where ϑ is a vector of eigenvalues of k. length. Once the family-wise error rate α is identified, one can retrieve the adjusted and correct α for each test, i.e., $\alpha_{T}$ as $\alpha_{T}=\alpha /T$. In our specific case, with k=43, T was 39.97, with a final $\alpha_{T}$. of 1.25^−03^. Accordingly, one can robustly reject the null hypothesis whenever the raw test *p* < $\alpha_{T}$. Correcting for multiplicity avoids the false rejection of 21 null hypotheses over a total of 122 tests. 

## 3. Results

### 3.1. Sociodemographic Characteristics of the Sample

The sample was similarly split into males (46%) and females (54%). Other sociodemographic data, including data regarding age ranges, education, and geographical area of residence, are detailed in Table 2. Participants were asked about their meat consumption habits and their intention to eat less meat. Eight percent of the sample declared that they do not eat meat, mainly women (i.e., 80%) (χ^2^ = 12.033; *p* < 0.01, that is to say, sex and non-meat consumption habits are associated. The chi-squared test is applied for testing independence between these two categorical variables). Ethics is the main reason for not consuming meat: 31% (of those respondents who stated that they do not eat meat) do not consume meat because intensive livestock production damages the environment, 38% because it is not animal welfare-friendly, 19% because of health-related reasons, and 12% because they do not like meat. Of those who eat meat, 37% stated their intention to reduce meat consumption, of which 56% were females (χ^2^ = 14.403; *p* < 0.01, therefore, sex and the intention to reduce meat consumption are associated). Reasons for decreasing meat consumption were equally shared between ethical and health issues: 25% intends to reduce meat consumption because intensive farming damages the environment, 26% because it is not animal welfare friendly, and 44% because meat has a negative impact on human health. Only a residual 4% cited the reason “meat is expensive”. No significant differences were found within age, sex, education, or place of residence.

### 3.2. The Change of Consumers’ Perception after Positive Information Provisioning 

In this section, the change in agreement for each of the six statements after the level II of information is analyzed (Table 1). After the provisioning of the first level of information (Level I), scores were higher for statements related to beliefs concerning positive externalities rather than intrinsic attributes: means of the three former affirmations resulted above the midpoint (i.e., above 3 on the Likert scale), with the protection of natural resources as the most valued attribute, whereas the rating means concerning beliefs related to the intrinsic attributes were below the Likert scale’s median, with the statement regarding the nutritional content of the cultured meat having the lowest score. 

To assess whether additional information affected respondents’ perception, the difference in means between the two different levels of information was tested for each statement (Δμ = (μ(II) − μ(I)), where μ indicates the mean) with respect to the whole sample. Results from the Wilcoxon rank test clearly awarded significant differences to the mean scores of all six statements, between the two levels of information. The highest differences were recorded for two affirmations with regards to consumers’ beliefs concerning the intrinsic attributes, namely “safety” and “nutrients”, whose mean differences are 0.4 and 0.5, respectively. Within the intrinsic group, “flavor” featured the smaller mean difference, i.e., 0.3. Concerning the beliefs related to the positive externalities, “sustainability” demonstrated the most substantial difference—0.3—representing the third attribute in absolute mean-difference terms. “Animal welfare” and “food security” followed, with a delta of 0.2 each. However, when observing the score-means, beliefs related to the intrinsic attributes still demonstrated lower rankings than positive externalities, even after the provisioning of the second level of information (we indicate the median as the statistical tests refer to the rank. However, for the sake of readability, we consider the mean value when commenting on the results).

For deepening the understanding, sociodemographic variables (i.e., sex, age, education, place of residence), consumption habits (meat/non-meat consumer, intention/no intention to reduce meat consumption), and familiarity with cultured meat were considered to deconvolute to what extent groups’ perception was affected by positive information. For the sake of simplicity, categories regarding age and education were gathered in more homogeneous groups—over 45 years old (n = 244) versus under 45 years old (n = 281)—and higher educated respondents, including university students and those who hold a university degree (n = 360) versus respondents who are not higher educated (n = 165). Table 3 shows that, after detailed and positive information provisioning, the agreement of almost all of the groups significantly increased to higher scores for all the statements. A notable exception when evaluating the beliefs concerning the positive externalities is represented by the “non-meat consumer” group: for them, the mean differences of “sustainability”, “animal welfare”, and “food security” are not statistically significant. The perception of those in the “18–44 years old”, “non-meat consumer”, “female”, and “academic degree and university students” groups was most affected by positive information concerning nutrition, with a delta generally above 0.6; perception of “non-meat consumer” and “female” groups changed most in terms of the intrinsic “safety” attribute (i.e., generally above 0.50); while “non-familiar with cultured meat” and “over 44 year’s old” groups’ perception was most greatly impacted in terms of the externality concerning “sustainability”, as differences resulted in around 0.5. Segmentation according to the place of residence was non-significant for all statements, hence the results are not reported in Table 3 (data are available upon request).

Briefly, while we can confirm that positive information on cultured meat affected respondents’ perception in the direction of the given information when the whole sample is considered, this is not always the case when subgroups were investigated. However, given the meagre number of subgroups showing non-significant differences, one could say that the provision of positive information certainly affected respondents’ perception towards a higher agreement with the six statements. 

### 3.3. The Change in Consumers’ WTT, WTB, and WTP after Positive Information Provisioning 

Sensitivity to information was also tested with reference to WTT, WTB, and WTP (Table 4). After the first level of information, 77% of the sample stated to be willing to try, 66% willing to buy, and 27% willing to pay a premium price (including WTP a +10%, +20%, and +30%) for cultured meat. After provisioning the level II of information, the change in WTT was not significant. 

WTB and WTP a premium price for cultured meat significantly increased, with their frequencies increasing by 7% and 27%, respectively. As far as the three decomposed options for WTP a premium, the WTP a 20% premium price rose significantly—by 36%—whereas the WTP a 10% and a 30% premium price were not significantly affected by the level II of information (Table S1 and Table S2).

Regarding the whole sample, given the significant increase in WTB and WTP a premium, one could say that some facets of acceptance have been positively affected by the information. The sample segmentation confirmed that none of the groups’ WTT was significantly affected by the provided information (Table 5). As far as WTB, information impacted meat consumers’ acceptance only, meanwhile information increased the WTP a premium price of the majority of the groups, namely “female”, “male”, “intend to reduce meat consumption”, “academic degree and current university student”, “meat consumer”, “18–44 years old”, and “familiar with cultured meat”.

## 4. Discussion

The preliminary evidence, as the object of the present study, is that respondents’ perceptions were particularly affected by additional information concerning two intrinsic attributes—nutritional aspects and safety of cultured meat. Respondents’ perception was less affected by additional information concerning externalities, probably because of the high level of agreement with the related statements even before the provision of positive information, while, with regards to the flavor attribute, it is likely that consumers need to be reassured in ways other than information provisioning. Indeed, sensory tests are crucial for acceptance of a novel food [73], in particular for meat substitutes such as cultured meat [39], since consumers are not willing to compromise a great deal on the taste of meat substitutes [37] and need the so-called “familiar flavor” to reduce food neophobia [74].

Segmenting according to sociodemographics and meat consumption habits revealed that perception of almost all groups is affected by additional information. The main exception is represented by non-meat consumers and, to a lesser extent, by “over 44 years old” and “no academic degree and no university student” respondents. For all the remaining groups, the registered change in perception is consistent with the type of information provided. This finding is in line with those of previous studies, according to which acceptance is sensitive to information provision [37], and the direction of the information provided can address the explicit attitude towards the unfamiliar object accordingly [38]. 

Vegetarians’ perception of cultured meat sustainability, animal welfare, and food security implications were unaffected by additional information. An explanation for this could be that vegetarian respondents were informed about the negative externalities of intensive livestock even before the provision of the positive information about cultured meat, as was shown by the introductory section of the questionnaire, where they declared that they do not consume meat mainly for ethical-related reasons. This is also consistent with the literature whereby non-meat consumers are well aware of the impact of intensive livestock farming on the environment and animal welfare [75].

Likewise, “no academic degree and no university student” and “over 44 years old” respondents were indifferent to information with regard to food security and flavoring in the former, and to food security in the latter. Again, such results are consistent with previous research reporting that people holding a higher education degree are more likely to engage in analytical thinking [76] rather than emotional attitudes, possibly making them more available to new food scenarios than lower educated consumers; conversely, age is negatively correlated with new experiences, implying that older people prefer to maintain established habits [77], which can be translated into a cautious attitude towards cultured meat.

Apart from the aforementioned exception, perceptions of nutritional, safety, and sustainability attributes of cultured meat were those most affected by additional information. Nevertheless, depending on consumers’ characteristics, the perception changed radically. In light of this, marketers and, possibly, policymakers would have to deliver a set of tailored messages to engage with different audiences. Indeed, the results suggest that the 18–44 years old age range, females, highly educated, and non-meat consumers’ perceptions were particularly affected by additional information on nutritional characteristics; meanwhile, non-familiar with cultured meat and, again, females and non-meat consumers’ perceptions showed the largest change after additional information related to safety aspects was provided. Finally, additional information about the sustainability of cultured meat exerted the most influential consequences on the perception of those not familiar with cultured meat and over 44 years old.

A point to stress, which proved to be consistent with what was reported in [45], is the pivotal importance of information for those without previous knowledge of the product, i.e., “not familiar with cultured meat”. They were amongst those that were the most affected by information on two externalities and one intrinsic attribute. The former two, “sustainability” and “food security”, confirm the importance of supporting the information concerning these positive externalities, as they are not quickly and directly identified as being related to cultured meat. Referring to beliefs concerning the intrinsic attribute, i.e., safety, it seems that for those unfamiliar with this product, it is of crucial importance to explain how the product is not harmful to human health. In fact, previous research on the sense of unfamiliarity with novel technologies, such as genetically modified organisms (GMOs) [78] to which cultured meat is associated [33], showed that unfamiliarity leads to a lack of trust [34], uncertainty, and concerns over potential adverse long-term consequences [34,47]. 

As far as the acceptance of cultured meat, none of the groups showed a significant variation in their willingness to try between the two levels of information. This is possibly related to the fact that respondents of this survey showed a high rate of willingness to try even before the provision of the second level of information, suggesting that consumers were already positive and interested in the product, and that the so-called yuck factor (i.e., the disgust that consumers might feel at the idea of consuming cultured meat) is not prominent. This result is consistent with Eurobarometers’ findings [79], according to which Italians were among the citizens in Europe who most favored cultured meat as an alternative to animal slaughtering. Another interpretation, in line with the results reported in [34,73], could be that willingness to try very much depends upon stimuli other than information. Indeed, as much as the perception was significantly affected by information, many groups did not show a significant variation regarding their willingness to try. If this is the case, new approaches are required to address “the underlying worldviews, fears, and conspirational mindsets that are associated with people resistance (p.144)” [30]. In this regard, the authors of [28] believe that the analysis of the attitude towards novel foods should start from the “understanding of the food identity profile of the members of the population of interest … to tap the psychological variables linked to the system of values that drive food choices” (p.10).

The findings also show that, after the positive information provisioning, the willingness to buy of meat consumers, who represent the lion share of the total sample, increased; conversely, non-meat consumers, over 44 years old respondents, those not holding a higher education degree, those with no intention of reducing their meat consumption and are not familiar with cultured meat did not show any change in their willingness to buy.

Regarding the last qualitative question posed in Section 2, i.e., (iii) whether the change in the perception, prompted by information provision, is predictive of a change in the acceptance, the results appeared mixed. On the one hand, despite the enhancement in perception between the two levels of information, the magnitude of such a change, or the change per se, seems not to be predictive of a positive shift in the intention to try the product. On the other hand, however, both the intention to purchase and to pay a premium price of the sample as a whole significantly rose, pointing to the existence of some predictive power of information on consumer attitudes over food choice. However, this represents a qualitative exercise, thus it presents an opportunity for future research on this topic.

The present research conveys interesting insights with regard to non-meat consumers and females. The main share of non-meat consumers provided ethical-related reasons as to why they did not consume meat. While they were shown to be familiar with the positive externalities of cultured meat, the opposite is true regarding its intrinsic attributes, as shown by their high sensitivity to two out of three intrinsic attributes, namely those related to nutrients and safety. Nevertheless, they were not willing to try, buy, or pay a premium price for cultured meat in either of the two levels of information in the questionnaire. This confirms the findings of [40], in which it was argued that those who do not consume meat for ethical reasons may support the product in terms of its improvement to both animal and environmental conditions but they are not willing to participate in its consumption. This coincides with the debate surrounding cultured meat among vegetarians. Indeed, most of them point out that cells used for cultured meat production are derived from animals, hence qualifying cultured meat as real meat [80]. Therefore, vegetarians prefer meat substitutes, such as plant-based burgers, rather than cultured meat [41]. Another point is that those vegetarians who do not eat meat for health-related reasons can represent an interesting field for future research because cultured meat would represent an appealing opportunity for consuming meat free from negative, health-related side effects (e.g., high cholesterol) and would open a further niche of potential customers.

With regards to the “females” group, this represents an interesting case of a changing perception after information provisioning. Indeed, women attributed lower scores to the six statements concerning both intrinsic attributes and externalities after receiving the first level of information, when compared to men. This was also the case for their willingness to pay a premium. The cautious perception shown by females towards novel food and genetically modified products is confirmed by several studies: [40] found that women display more negative attitudes than males towards cultured meat, and [81] confirmed that men are more willing to try cultured meat than women. Studies [82,83] showed how females are more reluctant to accept highly engineered food, particularly genetically modified products, whereas findings regarding insect-based food cemented the greater “food-neophobic attitude” of female subjects when compared to male [52]. Nevertheless, our survey showed that the delta resulting between the first and the second level of information provision is always higher for females than males, for the beliefs concerning both the externalities and intrinsic attributes, and for the willingness to pay a premium. Thus, enriching the informational background profoundly affected females’ perception and acceptance, entirely bridging the initial gap between the females and males.

Briefly, respondents of this survey can be classified according to two typologies: (a) those whose perception and their acceptance (in terms of WTB and WTP) of cultured meat were both significantly affected by positive information and (b) those whose perception was affected but their acceptance of cultured meat is partially, or not at all, affected by information. Within the former typology, meat consumers are featured, whereas the latter typology is represented by several subgroups (i.e., “no intention to reduce meat consumption”, “no academic degree and no university students”, “non-meat consumer”, “over 44 years old”, and “not familiar with cultured meat”). This typology should be further investigated to understand what remains uncomfortable for these groups when it comes to cultured meat. It is likely that the cautious attitude of this consumer typology is related to the food culture of the country where the survey took place—Italy—in which food quality is mainly equated to the naturalness of the product [84]. If so, positive information alone will not be sufficient to overcome aversion to cultured meat and different approaches will be necessary to change the present consumers’ mindset and system of values that prevent them from accepting cultured meat.

One main limitation of the presented study lies in the potential bias of the results, as consumers tend to overestimate their willingness to pay when in a hypothetical context [85]. To this extent, a lot of information on consumers’ acceptance towards cultured meat will be gained in the near future, when the US market will be (most likely) the first to make cultured meat available to consumers. A second limitation is that the sample, when compared to the census population, is younger (only 8% of the sample was in the >65 age category, compared to 22% in the census population) and better educated (95% of the sample reports holding a post-secondary diploma compared to 42% of the census population). This high level of education is likely due to the scarce representation of the elderly in the sample. Hence, the sample cannot be considered fully representative of the population, and the percentage of respondents willing to try cultured meat is biased by the share of young people in the sample; however, it includes consumers who are more likely to be involved in future novel food scenarios.

Future research on the impact of information on consumers’ acceptance of cultured meat shall cover a wide range of issues. Hitherto, surveys tended to highlight the positive characteristics of cultured meat, disregarding critical aspects that could arise [86] (e.g., the massive use of energy in the case of large-scale production, the economic sustainability of livestock farmers, and the agricultural substitution of surfaces that were dedicated to meat-producing animals, among many others). Accordingly, evaluating consumers’ perceptions regarding the trade-off between positive and negative, such as price, or uncertain characteristics of cultured meat is of interest, particularly for policymakers. Moreover, assuming that meat consumers are those who are the most interested in cultured meat, an interesting perspective could be the analysis of the reasons why some consumers eat meat and are not interested in their reducing meat consumption. Indeed, some people eat meat for reasons other than taste, such as athletes who follow high-protein diets. For them, cultured meat would represent a compromise between their nutritional needs and ethical beliefs. This seems to alleviate the “meat paradox”: cultured meat could provide a new balance between ethics (e.g., animal wellbeing and environmental concerns) and actual consumption behaviors.

The latest aspect concerns the sources providing the information and their diverse impact on consumers’ acceptance. Independent third-party bodies, including universities, may induce a more significant impact than private companies; in light of this, the communication of European Food Safety Authority approval of cultured meat, if any, would represent a strong guarantee to the consumers.

## 5. Conclusions

This research assessed for which beliefs concerning intrinsic attributes and positive externalities, the provision of positive information represents a sufficient tool for affecting the perception and acceptance of cultured meat on a panel of Italian consumers. Changes in perception and willingness to try, buy, and pay were assessed by measuring the variation before and after the provision of positive information related to the product. The results showed that perception is significantly affected when the information concerns safety and nutritional characteristics, whereas the opposite occurs regarding the product flavour. Furthermore, findings revealed that, while the willingness to buy increases after providing positive information, the willingness to try does not. Indeed, willingness to try depends upon further stimuli other than information, suggesting a deeper analysis of the food profile, and the values underlying it, of the population of interest. Therefore, information on intrinsic attributes and positive externalities of the cultured meat would have to be combined with different approaches for further enhancement of consumers’ perception and acceptance.

## Figures and Tables

**Table 1:** Beliefs concerning positive externalities and intrinsic attributes ^a^.

Description	Label	Median	μ_(I),(II)_	Std. Dev.	Δμ (μ_(II)_ − μ_(I)_)	Wilcoxon	*p* ^b^
	Beliefs concerning positive externalities						
Cultured meat will contribute to preserve natural resources	Sustainability (I)	4	3.5	1.3	0.3	−8.50	<*p*-adj
	Sustainability (II)	4	3.8	1.3			
Cultured meat is animal welfare friendly	Animal Welfare (I)	3	3.1	1.4	0.2	−6.58	<*p*-adj
	Animal Welfare (II)	4	3.3	1.4			
Cultured meat will contribute to alleviate hunger in developing countries	Food Security (I)	3	3.1	1.4	0.2	−6.46	<*p*-adj
	Food Security (II)	4	3.4	1.4			
	Beliefs concerning intrinsic attributes						
A cultured meat burger will be as tasty as a conventional burger	Flavor (I)	2	2.5	1.2	0.3	−7.38	<*p*-adj
	Flavor (II)	3	2.8	1.3			
A cultured meat burger will be more nutrient than a conventional burger	Nutrients (I)	2	2.4	1.1	0.5	−10.23	<*p*-adj
	Nutrients (II)	3	2.9	1.3			
Cultured meat is safe	Safety (I)	3	2.8	1.2	0.4	−10.54	<*p*-adj
	Safety (II)	3	3.2	1.3			

^a^ Evaluation based on five-point Likert scale: 1 = I do not agree at all; 5 = I definitely agree. ^b^ Whenever the actual *p*-value is smaller than the adjusted *p*, the H_0_ can be rejected.

**Table 2:** Sociodemographic characteristics of the sample.

Variable	%	n.
Age		
<25	19.40%	102
25–45	34.10%	179
46–65	38.30%	201
>65	8.20%	43
Total Age	100.00%	525
Sex		
Female	54.00%	282
Male	46.00%	243
Total Sex	100.00%	525
Education		
Degree and PhD	53.10%	279
Secondary school	42.30%	222
High school	4.40%	23
Primary school	0.20%	1
Total Education	100.00%	525
Place of residence		
North	70.50%	370
Centre	15.20%	80
South	14.30%	75
Total Place of residence	100.00%	525

**Table 3:** Medians, average scores, and significance level of score differences between the level II and the level I of information for beliefs on positive externalities and intrinsic attributes per group

**Groups**	**Obs.**	**Sustainability (II) - Sustainability (I)**					**Animal Welfare (II) - Animal Welfare (I)**					**Food Security (II) - Food Security (I)**				
	**N.**	**Median ^a^**	**μ_1_**	**Δ**	**Wilcoxon**	***p*^b^**	**Median ^a^**	**μ_1_**	**Δ**	**Wilcoxon**	***p*^b^**	**Median ^a^**	**μ_1_**	**Δ**	**Wilcoxon**	***p*^b^**
Female	282	4	3.4	0.4	−7.05	<p-adj	3	3.1	0.3	−5.12	<p-adj	3	3.1	0.3	−5.61	<p-adj
		4	3.8				4	3.3				4	3.3			
Male	243	4	3.5	0.3	−4.79	<p-adj	3	3.1	0.2	−4.14	<p-adj	3	3.2	0.2	−3.45	<p-adj
		4	3.8				4	3.3				4	3.4			
Intention to Reduce Meat Consumption	182	4	3.5	0.4	−5.19	<p-adj	3	3.2	0.3	−4.71	<p-adj	3	3.2	0.2	−3.34	<p-adj
		4	3.9				4	3.5				4	3.4			
No intention to Reduce Meat Consumption	303	4	3.4	0.3	−6.73	<p-adj	3	3	0.2	−4.56	<p-adj	3	3.1	0.2	−5.13	<p-adj
		4	3.8				3	3.2				4	3.3			
Academic Degree and Current University Student	360	4	3.7	0.3	−6.62	<p-adj	3	3.2	0.2	−5.63	<p-adj	3	3.2	0.3	−5.76	<p-adj
		4	4				4	3.5				4	3.5			
No Academic Degree and no University Student	165	3	3	0.4	−5.34	<p-adj	3	2.8	0.2	−3.44	<p-adj	3	2.9	0.2	−3.01	>p-adj
		4	3.4				3	3				3	3.1			
Meat Consumer	485	4	3.5	0.3	−8.46	<p-adj	3	3.1	0.3	−6.48	<p-adj	3	3.2	0.2	−6.04	<p-adj
		4	3.8				4	3.3				4	3.4			
Non-Meat Consumer	40	4	3.7	0.2	−1.21	>p-adj	3	3.2	0.1	−1.17	>p-adj	3	3	0.3	−2.5	>p-adj
		4	3.9				3	3.4				3	3.3			
18–44 years old	281	4	3.7	0.2	−4.53	<p-adj	3	3.2	0.2	−4.5	<p-adj	3	3	0.3	−5.93	<p-adj
		4	4				4	3.4				4	3.3			
Over 44 years old	244	3	3.2	0.4	−7.41	<p-adj	3	3	0.3	−4.79	<p-adj	4	3.3	0.2	−3.02	>p-adj
		4	3.6				3	3.2				4	3.4			
Non-Familiar with Cultured Meat	180	3	3.1	0.5	−6.39	<p-adj	3	2.9	0.3	−3.89	<p-adj	3	2.9	0.3	−4.37	<p-adj
		4	3.6				3	3.2				3.5	3.2			
Familiar with Cultured Meat	345	4	3.7	0.3	−5.7	<p-adj	3	3.2	0.2	−5.32	<p-adj	3	3.3	0.2	−4.75	<p-adj
		4	3.9				4	3.4				4	3.5			
**Groups**	**Obs.**	**Flavor (II) - Flavor (I)**					**Nutrients (II) - Nutrients (I)**					**Food Safety (II) - Food Safety (I)**				
	**N.**	**Median ^a^**	**μ_ι_**	**Δ**	**Wilcoxon**	***p*^b^**	**Median ^a^**	**μ_1_**	**Δ**	**Wilcoxon**	***p*^b^**	**Median ^a^**	**μ_1_**	**Δ**	**Wilcoxon**	***p*^b^**
Female	282	3	2.5	0.3	−5.85	<p-adj	2	2.3	0.6	−8.25	<p-adj	3	2.7	0.5	−8.46	<p-adj
		2	2.8				3	2.9				3	3.2			
Male	243	3	2.5	0.2	−4.47	<p-adj	3	2.5	0.4	−6.1	<p-adj	3	2.8	0.4	−6.32	<p-adj
		3	2.7				3	2.9				3	3.2			
Intention to Reduce Meat Consumption	182	3	2.7	0.3	−4.36	<p-adj	3	2.5	0.5	−6.21	<p-adj	3	2.8	0.5	−6.52	<p-adj
		3	3				3	3				3	3.2			
No intention to Reduce Meat Consumption	303	2	2.3	0.3	−5.77	<p-adj	2	2.3	0.5	−7.41	<p-adj	3	2.7	0.4	−7.61	<p-adj
		3	2.6				3	2.8				3	3.1			
Academic Degree and Current University Student	360	3	2.6	0.3	−6.72	<p-adj	2	2.4	0.6	−9.48	<p-adj	3	2.9	0.5	−9.55	<p-adj
		3	2.9				3	3				4	3.4			
No Academic Degree and no University Student	165	2	2.3	0.2	−3.15	>p-adj	2	2.4	0.3	−4.14	<p-adj	2	2.4	0.4	−4.83	<p-adj
		3	2.5				3	2.7				3	2.8			
Meat Consumer	485	2	2.5	0.3	−7.17	<p-adj	2	2.4	0.5	−9.64	<p-adj	3	2.7	0.4	−10.01	<p-adj
		3	2.7				3	2.9				3	3.2			
Non-Meat Consumer	40	3	3.1	0.2	−1.69	>p-adj	3	2.6	0.6	−3.57	<p-adj	3	2.9	0.6	−3.33	<p-adj
		3.5	3.3				3	3.2				4	3.4			
18–44 years old	281	3	2.6	0.3	−5.36	<p-adj	2	2.4	0.6	−8.74	<p-adj	3	2.9	0.5	−8.35	<p-adj
		3	2.8				3	3				4	3.3			
Over 44 years old	244	2	2.4	0.3	−5.04	<p-adj	3	2.4	0.4	−5.53	<p-adj	3	2.6	0.4	−6.54	<p-adj
		3	2.7				3	2.8				3	3			
Non-Familiar with Cultured Meat	180	2	2.2	0.3	−4.19	<p-adj	2	2.1	0.5	−5.53	<p-adj	2	2.4	0.5	−7.06	<p-adj
		3	2.5				3	2.7				3	2.9			
Familiar with Cultured Meat	345	3	2.7	0.3	−6.09	<p-adj	3	2.5	0.5	−8.67	<p-adj	3	3	0.4	−7.9	<p-adj
		3	2.9				3	3				4	3.4			

^a^ Please note that the range for each statement and for each point in time is always equal to four given that at least one respondent always scored 1 and 5 on the provided Likert scale. ^b^ Whenever the actual *p* is smaller than the adjusted *p*, the H_0_ can be rejected.

**Table 4:** Willingness to pay (WTP), try (WTT), and buy (WTB). Median, means, and significance of differences between the level II and the level I of information.

Variable *	Obs. ^b^	Median	Mean	Mean Difference	Wilcoxon	*p* ^c^
WTP_Premium (I) ^a^	348	0	0.40	0.07	−5.00	<p-adj
WTP_Premium (II) ^a^	371	0	0.47			
WTP_ + 30% (I)	26	0	0.07	0.01	−2.65	>p-adj
WTP_ + 30% (II)	33	0	0.09			
WTP_ + 20% (I)	53	0	0.15	0.04	−3.40	<p-adj
WTP_ + 20% (II)	72	0	0.19			
WTP_ + 10% (I)	60	0	0.17	0.02	−0.91	>p-adj
WTP_ + 10% (II)	71	0	0.19			
WTP_zero (I)	55	0	0.16	−0.02	−1.22	>p-adj
WTP_zero (II)	50	0	0.13			
WTP_Less (I)	65	0	0.19	−0.04	−3.27	<p-adj
WTP_Less (II)	56	0	0.15			
WTT (I)	525	1	0.77	0.02	−2.07	>p-adj
WTT (II)	525	1	0.79			
WTB (I)	525	1	0.66	0.04	-3.68	<p-adj
WTB (II)	525	1	0.71			

* Since all the listed variables are dicothomic, i.e., taking values of either 0 or 1, each variable’s range is always 1. ^a^ WTP_Premium includes WTP a +10%, +20%, and +30% premium price; WTP_Less includes a −10%, −20%, and −30% price compared to the conventional price. ^b^ Please see Tables S1 and S2 for the descriptive statistics of the listed variable. ^c^ When the actual *p*-value is smaller than the adjusted *p*-value, the H_0_ can be rejected.

**Table 5:** Medians, average scores, and significance level of score differences between the level II and the level I of information for WTT, WTB, and WTP a premium per group

Groups	Obs.	WTT (II) - WTT (I)					WTB (II) - WTB (I)					WTP_Premium (II) - WTP_Premium (I)				
	N.	Median	μ_ι_	Δ	Wilcoxon	*p* ^a^	Median	μ_ι_	Δ	Wilcoxon	*p* ^a^	Median	μ_ι_	Δ	Wilcoxon	*p* ^a^
Female	282	1	0.8	0.02	−1.73	>p-adj	1	0.7	0.06	−3.02	>p-adj	0	0.4	0.1	−3.8	<p-adj
		1	0.8				1	0.7				1	0.5			
Male	243	1	0.8	0.01	−1.13	>p-adj	1	0.7	0.03	−2.11	>p-adj	0	0.4	0.06	−3.32	<p-adj
		1	0.8				1	0.7				0	0.4			
Intention to Reduce Meat Consumption	182	1	0.8	0.02	−1.34	>p-adj	1	0.7	0.05	−2.83	>p-adj	0	0.5	0.11	−3.9	<p-adj
		1	0.8				1	0.8				1	0.6			
No intention to Reduce Meat Consumption	303	1	0.8	0.02	−1.39	>p-adj	1	0.7	0.04	−2.56	>p-adj	0	0.5	0.02	−3.15	>p-adj
		1	0.8				0	0.7				1	0.5			
Academic Degree and Current University Students	360	1	0.8	0.02	−1.73	>p-adj	1	0.7	0.04	−2.84	>p-adj	0	0.4	0.08	−4.43	<p-adj
		1	0.9				1	0.8				1	0.5			
No Academic Degree and no University Students	165	1	0.6	0.02	−1.13	>p-adj	1	0.5	0.06	−2.36	>p-adj	0	0.3	0.06	−2.33	>p-adj
		1	0.7				1	0.6				0	0.4			
Meat Consumer	485	1	0.8	0.02	−1.89	>p-adj	1	0.7	0.05	−3.57	<p-adj	0	0.4	0.09	−5	<p-adj
		1	0.8				1	0.7				0	0.5			
Non-Meat Consumer	40	0	0.5	0.02	−1	>p-adj	0	0.5	0.03	−1	>p-adj	1	0.8	−0.04	0	>p-adj
		0.5	0.5				0	0.5				1	0.8			
18–44 years old	281	1	0.8	0.02	−1.51	>p-adj	1	0.7	0.04	−2.52	>p-adj	0	0.4	0.1	−4.6	<p-adj
		1	0.8				1	0.8				1	0.5			
Over 44 years old	244	1	0.7	0.01	−1.41	>p-adj	1	0.6	0.05	−2.68	>p-adj	0	0.4	0.03	−2.11	>p-adj
		1	0.7				1	0.6				0	0.4			
Non-Familiar with Cultured Meat	180	1	0.7	0	0	>p-adj	1	0.6	0.06	−2.84	>p-adj	0	0.3	0.06	−2.53	>p-adj
		1	0.7				1	0.6				0	0.3			
Familiar with Cultured Meat	345	1	0.8	0.03	−2.71	>p-adj	1	0.7	0.03	−2.45	>p-adj	0	0.5	0.09	−4.32	<p-adj
		1	0.8				1	0.7				1	0.6			

^a^ Whenever the actual *p*-value is smaller than the adjusted *p*, the H_0_ can be rejected.

## Supplementary Materials

The following are available online at https://www.mdpi.com/2076-2615/10/4/656/s1; Figure S1. Graphical representation of the questionnaire structure; Figure S2. Graphical representation of the questionnaire content; Table S1. WTT, WTB and their variations between the level II and the level I of information; Table S2. WTP and variation between the level II and the level I of information.
