# Supplementary material for: To What Extent Are Consumers’ Perception and Acceptance of Alternative Meat Production Systems Affected by Information? The Case of Cultured Meat
Source: Animals (Basel). 2020 Apr 10;10(4):656. doi: 10.3390/ani10040656 (PMC7223365; doi:10.3390/ani10040656)
Supplement: Supplementary file 1 [file animals-10-00656-s001.pdf]

Figure S1. Graphical representation of the questionnaire structure

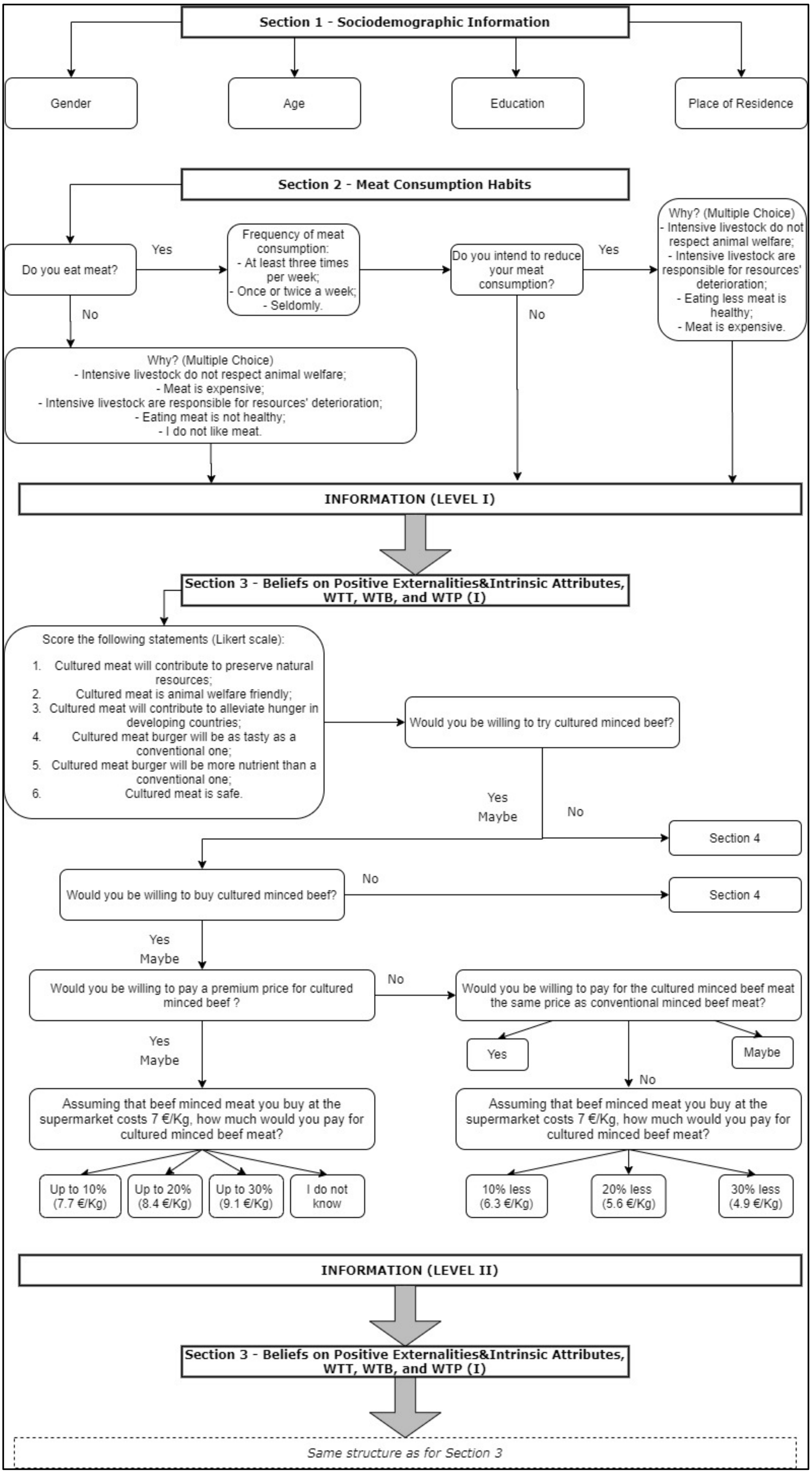

Figure S2. Graphical representation of the questionnaire content

INFORMATION (LEVEL I)

"Cultured beef is created by harvesting muscle cells from a living cow. Researchers at the Maastricht University (culturedbeef.org) state that the cells from a single cow would produce 175 million quarter-pounders. Conventional farming methods would require 440,000 cows. The product should not be confused with meat substitutes, such as tofu, because it is real meat, the same as the one produced according to the current intensive livestock production system. Cultured meat is not yet available on the market".

1) Cells are extracted from the living animal.

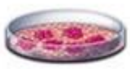

3) Cells, under stimulation, are grown into muscle tissues.

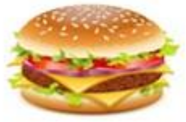

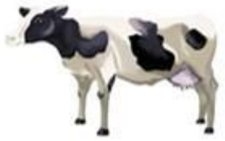

2) Cells are nurtured and melted.

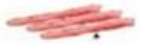

4) Muscle tissues are minced and mixed with fats (they are cultured too) and shaped.

Source: Author's elaboration on culturedbeef.org, Maastricht University

INFORMATION (LEVEL II)

"Two-thirds of agricultural land is used for livestock production, which is responsible for approximately 18% of the greenhouse gas (GHG) emissions (1). FAO estimates that the rising per capita income in developing countries will increase by 70% in forty years. It was shown (2) that the production of cultured meat would lead to a 98.8% reduction in GHG emissions, a 99.7% reduction in land use and a 94% reduction in water use compared to the production of traditional meat. The production of cultured meat would also prevent diseases, such as mad cow disease, and microbiological infections, such as *Salmonella* infections, due to the occurrence of fewer contacts between animals and humans. In addition, the fat composition of the meat can be improved, for example, by enriching the meat with omega-3 fatty acids."

(1) FAO (2012). Livestock and landscapes, pp. 1-4 Rome http://www.fao.org/docrep/018/ar591e/ar591e.pdf;
FAO (2011). World livestock 2011, pp. 1-130 Rome http://www.fao.org/docrep/014/i2373e/i2373e00.htm;
(2) Tuomisto, H. L., Ellis, M. J., & Haastrup, P. (2014, October). Environmental impacts of cultured meat: alternative production scenarios. In Proceedings of the 9th International Conference on Life Cycle Assessment in the Agri-food Sector (pp. 8-10);
Tuomisto, H. L., & Roy, A. G. (2012, October) Could cultured meat reduce environmental impact of agriculture in Europe. In 8th international conference on LCA in the Agri-food sector, Rennes, France (pp. 2-4)

Table S1. WTT, WTB and their variations between the level II and the level I of information

	**Frequency**	**Share on Total Sample**	**Frequency**	**Share on Total Sample**	**Frequency**	**ΔPer cent**
	WTT (I)		*WTT (II)*		*ΔWTT*	
**No**	121	23%	112	21%	-9	-7
**Yes**	404	77%	413	79%	9	2
**Total**	525	100%	525	100%		
	WTB (I)		*WTB (II)*		*ΔWTB*	
**No**	177	33.7%	154	29.3%	-23	-13
**Yes**	348	66.3%	371	70.7%	23	7
**Total**	525	100.0%	525	100.0%		

Source: authors' elaboration

Table S2. WTP and variation between the level II and the level I of information

	WTP (I)		***WTP (II)***		**□*WTP***	
	Freq.	Share on Total Sample	Freq.	Share on Total Sample	Freq.	◎Per cent
**Premium Price (+10%)**	60	11.4	71	13.5	11	18
**Premium Price (+20%)**	53	10.1	72	13.7	19	36
**Premium Price (+30%)**	26	5	33	6.3	7	27
**Same Price**	55	10.5	50	9.5	-5	-9
**Lower Price (-30%)**	38	7.2	33	6.3	-5	-13
**Lower Price (-20%)**	15	2.9	10	1.9	-5	-33
**Lower Price (-10%)**	12	2.3	13	2.5	1	8
**"Maybe the same price"**	44	8.4	36	6.9	-8	-18
**"I don't know how much more I would pay for it"**	45	8.6	53	10.1	8	18
**Total Valid**	348	66.3	371	70.7	23	7
**No intention to try or buy cultured meat**	177	33.7	154	29.3	-23	-13
**Total**	525	100	525	100	-	-
